# Synthesis of ω-Chloroalkyl Aryl Ketones via C–C Bond Cleavage of *tert*-Cycloalkanols with Tetramethylammonium Hypochlorite

**DOI:** 10.3390/molecules29081874

**Published:** 2024-04-19

**Authors:** Natsumi Hanazawa, Masami Kuriyama, Kosuke Yamamoto, Osamu Onomura

**Affiliations:** Graduate School of Biomedical Sciences, Nagasaki University, 1-14 Bunkyo-machi, Nagasaki 852-8521, Japanmkuriyam@nagasaki-u.ac.jp (M.K.); kyamamoto@nagasaki-u.ac.jp (K.Y.)

**Keywords:** C–C bond cleavage, oxidation, *tert*-cycloalkanols, tetramethylammonium hypochlorite, ω-chloroalkyl aryl ketones

## Abstract

An oxidative C–C bond cleavage of *tert*-cycloalkanols with tetramethylammonium hypochlorite (TMAOCl) has been developed. TMAOCl is easy to prepare from tetramethylammonium hydroxide, and the combination of TMAOCl and AcOH effectively promoted the C–C bond cleavage in a two-phase system without additional phase-transfer reagents. Unstrained *tert*-cycloalkanols were transformed into ω-chloroalkyl aryl ketones in moderate to excellent yields under metal-free and mild reaction conditions.

## 1. Introduction

Chloro-substituted ketones are versatile building blocks in the synthesis of drug-candidate compounds [1,2,3]. Therefore, the development of an efficient synthetic method for chlorinated ketones is highly desirable. While a number of reliable protocols for the synthesis of chlorinated ketones have been developed [4,5,6,7], the straightforward introduction of a chlorine atom into the remote position of a carbonyl group is still challenging. Although the Friedel–Crafts reaction is one of the direct approaches for chlorinated carbonyl compounds, stoichiometric strong Lewis acids and moisture-sensitive acyl chlorides are required [8,9,10].

**Scheme 1:**
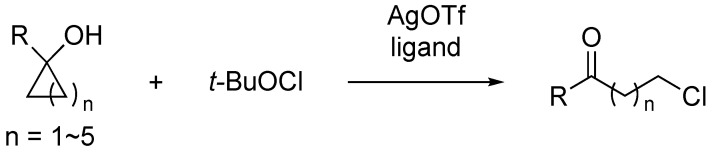
Regiospecific synthesis of carbonyl-containing alkyl chlorides with *t*-BuOCl.

**Scheme 2:**
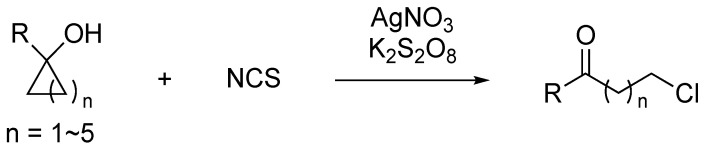
Regiospecific synthesis of carbonyl-containing alkyl chlorides with NCS.

**Scheme 3:**
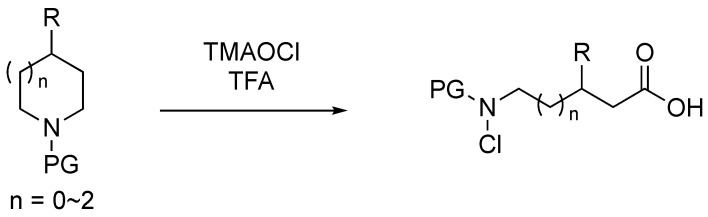
Our previous work: oxidative C–N bond cleavage of cyclic amines with TMAOCl.

**Scheme 4:**
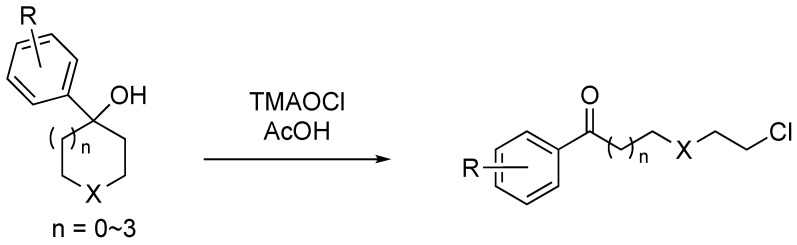
This work: oxidative C–C bond cleavage of *tert*-cycloalkanols with TMAOCl.

Alkoxy radicals are known as highly reactive species, involved in inert C(sp^3^)−H and C(sp^3^)−C(sp^3^) bond functionalization through 1,5-hydrogen atom transfer and β-scission reactions [11,12,13,14]. The ring-opening chlorination of cycloalkanols has proven to be an efficient strategy for the synthesis of distally chloro-substituted ketones through β-scission. *tert*-Cyclopropanols and cyclobutanols are useful precursors for β- and γ-chlorinated ketones since the ring-strain energies of these cycloalkanols are highly favorable for promoting the β-scission of the alkoxy radical species [15,16,17,18]. On the other hand, similar ring-opening reactions of unstrained cycloalkanols have also attracted attention as efficient synthetic methods for ω-functionalized ketones [19,20,21]. For example, Zhang and Qi reported the synthesis of ω-chloroalkyl aryl ketones from corresponding cycloalkanols using *t*-BuOCl as a Cl source (Scheme 1) [22]. The group of Zhu and Bao developed the ring-opening reaction with NCS (Scheme 2) [23]. In both cases, a precious transition-metal catalyst is required. In addition, there are not many examples of chlorinated-ketone synthesis from unstrained cycloalkanols [24,25,26,27,28]. Therefore, the development of alternatively greener and more efficient protocols is still desired. Hypochlorite salts are classical and versatile oxidants [29,30,31,32,33,34], and phase-transfer catalysts have often been employed in efficient hypochlorite-mediated oxidation methods [32,33,34]. Tetramethylammonium hypochlorite (TMAOCl) is easy to prepare from tetramethylammonium hydroxide by chlorine gas injection or the ion-exchange method [35,36]. In addition, TMAOCl is a unique hypohalite salt requiring no additional phase-transfer reagents. Recently, we disclosed the ring-opening chlorination of *N*-protected cyclic amines with the use of TMAOCl as an oxidant (Scheme 3) [37]. Herein, we describe the metal-free C(sp^3^)−C(sp^3^) bond cleavage of *tert*-cycloalkanols for the synthesis of ω-chloroalkyl aryl ketones with TMAOCl (Scheme 4).

## 2. Results and Discussion

### 2.1. Optimization of Reaction Conditions

We started the initial study with the use of 1-phenylcyclohexanol (**1a**) as a model substrate (Table 1). When **1a** (0.5 mmol) was treated with TMAOCl (1.5 equiv) and 35% HCl aq. (1.5 equiv) in CH_2_Cl_2_ at room temperature for 1 h, the desired ω-chloroalkyl aryl ketone **2a** was obtained in 47% yield (Table 1, entry 1). Among a series of acids tested, AcOH provided a superior result (entries 1–5). We further optimized the reaction conditions, and **2a** was obtained in a higher yield by employing 2.0 equiv of AcOH (entry 6). The yield of **2a** slightly decreased with the use of 2.25 equiv of AcOH (entry 7), and the use of 2.0 equiv of TMAOCl and AcOH led to an obvious decrease in yield (entry 8). When the reactions were carried out at higher reaction concentrations, the reaction outcomes were not affected (entries 9 and 10), and **2a** was isolated in 83% yield (entry 10). Extending the reaction time did not improve the yield of **2a** (entry 11). Other solvents such as ClPh, AcOEt, and MeCN were less suitable for the reaction, providing **2a** in lower yields (entries 12–14). A commercially available NaOCl·5H_2_O was found to be a less effective oxidant for this ring-opening reaction (entry 10 vs. entry 15). The addition of Me_4_NCl exhibited a positive effect on the reaction outcome [38,39], but the yield was slightly low, even with 2.0 equiv of NaOCl·5H_2_O, compared with that obtained from TMAOCl (entry 10 vs. entries 16 and 17). On the other hand, the reaction did not proceed well without an acid (entry 18). The reaction under the N_2_ atmosphere provided **2a** in 77% yield, indicating that the presence of oxygen did not have a significant effect on the reaction outcome (entry 19) [40].

### 2.2. Substrate Scope

**Scheme 5:**
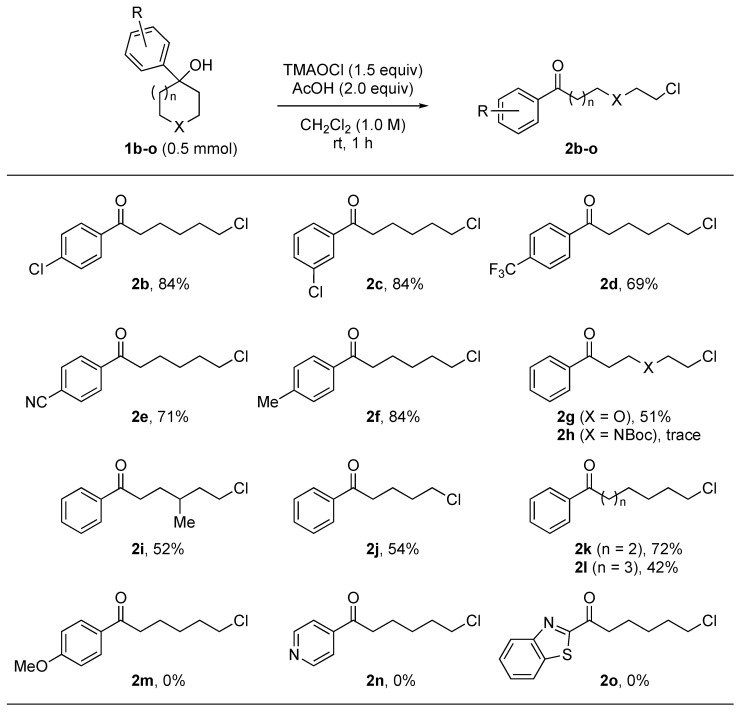
Substrate scope and limitations for C–C bond cleavage of *tert*-cycloalkanols. Reagents and conditions: **1** (0.5 mmol), TMAOCl (1.5 equiv), AcOH (2.0 equiv), CH_2_Cl_2_ (1.0 M), rt, 1 h. Isolated yields are shown.

With optimized conditions in hand, the scope and limitation of this reaction were investigated (Scheme 5). The substrate **1b** with a *p*-chloro group provided the desired product **2b** in 84% yield. Moreover, 1-(3-Chlorophenyl)cyclohexanol (**1c**) was converted into the corresponding product **2c** in a high yield, and 1-Phenylcyclohexanols, substituted with electron-withdrawing groups such as trifluoromethyl (**1d**) and cyano (**1e**) groups participated well in this ring-opening reaction, affording the corresponding products (**2d** and **2e**) in good yields. The substrate with an electron-donating group, such as a *p*-methyl group, provided the desired product **2f** in 84% yield. While the tetrahydro-4-pyranol derivative **1g** was successfully transformed into the corresponding product **2g** in a moderate yield, the *N*-Boc-4-piperidinol derivative **1h** afforded a trace amount of the desired product **2h**. The reaction of 4-methyl-1-phenylcyclohexanol **1i** led to the formation of **2i** in 52% yield. Furthermore, cycloalkanols with different ring sizes were converted into the desired products (**2j**–**2l**) in moderate to high yields. The substrates with *p*-methoxyphenyl (**1m**), 4-pyridyl (**1n**), and benzothiazolyl (**1o**) groups were not suitable substrates for the present reaction conditions, and most of the starting materials remained unreacted [41].

### 2.3. Scale-Up Experiment

**Scheme 6:**
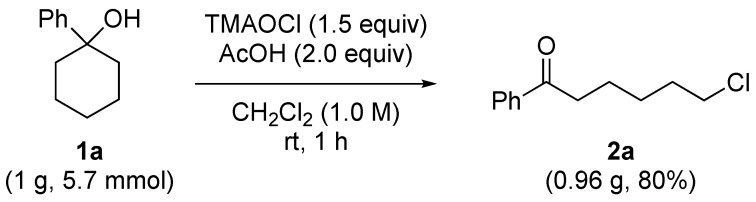
Scale-up experiment.

To evaluate the scalability of the present reaction, the reaction was carried out using 5.7 mmol of **1a** (Scheme 6). The desired product **2a** was obtained in 80% yield.

### 2.4. Control Experiment

A control experiment was conducted to gain insight into the details of the present transformation. The reaction of **1a** under the standard reaction conditions with 1.5 equiv of 2,2,6,6-tetramethylpiperidine-1-oxyl (TEMPO) provided the ring-opening product **2a** in 42% yield, and a TEMPO adduct was not detected in the crude reaction mixture by HRMS analysis. The previous literature reports proposed that the fragmentation of *tert*-alkyl hypochrolite proceeds through alkoxy radical formation [28,40,41,42,43,44]. However, the control experiment suggests that a radical process may be not necessarily involved in the present reaction.

## 3. Materials and Methods

### 3.1. Chemicals and Instruments

^1^H, ^13^C{^1^H}, and ^19^F NMR spectra were recorded with a JNM ECA400II spectrometer (JEOL, Tokyo, Japan) (400 MHz for ^1^H NMR, 100 MHz for ^13^C{^1^H} NMR, 376 MHz for ^19^F NMR). Chemical shift values are expressed in parts per million (ppm) relative to internal TMS (*δ* 0.00 ppm for ^1^H NMR) or CDCl_3_ (*δ* 77.0 ppm for ^13^C{^1^H} NMR). Abbreviations are as follows: s, singlet; d, doublet; t, triplet; and m, multiplet. Low- and high-resolution mass spectra (LRMS and HRMS) were recorded using a Xevo QTof MS system (Waters, Tokyo, Japan) using the electrospray ionization (ESI) method. Infrared (IR) spectra were recorded on a Spectrum Two spectrometer (Perkin–Elmer, Yokohama, Japan). Data are expressed as frequency of absorption (cm^−1^). The products were isolated by silica-gel column chromatography (Sfär Silica D Duo 60 µm, Biotage, Uppsala, Sweden). Commercially available chemicals were purchased from Sigma–Aldrich (Tokyo, Japan), Tokyo Chemical Industry Co., Ltd. (Tokyo, Japan) and FUJIFILM Wako Pure Chemical Corporation (Osaka, Japan), and used as received. A strong acid cation-exchange resin, Amberlite IR120B Na, was purchased from Organo Corporation (Tokyo, Japan). Aqueous tetramethylammonium hypochlorite (TMAOCl) was prepared from aqueous tetramethylammonium hydroxide (TMAOH) by ion-exchange method (14.0 wt% TMAOCl, 7.86 wt% available chlorine, pH = 11.5) [36]. Compounds **1b** [20], **1c** [45], **1d**–**1g** [20], **1h** [46], **1i** [47], **1j**–**1k** [20], **1l** [48], and **1m**–**1o** [20] were synthesized according to the reported methods.

### 3.2. General Procedure for the Oxidative C–C Bond Cleavage of tert-Cycloalkanol

*tert*-Cycloalkanol **1** (0.5 mmol) was added to a 9 mL vial, and then CH_2_Cl_2_ (0.5 mL), TMAOCl (0.673 mL, 0.75 mmol), and AcOH (60.0 mg, 57.2 μL, 1.0 mmol) were successively added at rt. After stirring for 1 h at the same temperature, the reaction mixture was extracted with AcOEt. The combined organic layers were dried over Na_2_SO_4_, filtered, and concentrated under reduced pressure. The residue was purified by silica-gel column chromatography to give the corresponding ω-chloroalkyl aryl ketones.

### 3.3. Procedure for the Scale-Up Experiment

1-Phenylcyclohexanol **1a** (5.7 mmol, 1.0 g) was added to a 20 mL vial, and then CH_2_Cl_2_ (5.7 mL), TMAOCl (0.67 mL, 8.55 mmol), and AcOH (684 mg, 0.65 mL, 11.4 mmol) were successively added at rt. After stirring for 1 h at rt, the reaction mixture was extracted with AcOEt. The combined organic layers were dried over Na_2_SO_4_, filtered, and concentrated under reduced pressure. The residue was purified by silica-gel column chromatography (hexane/AcOEt = 97:3) to produce 0.96 g of chloro-1-phenylhexan-1-one **2a** (4.56 mmol, 80%).

### 3.4. Preparation of Aqueous TMAOCl Solution

IR120B Na (0.5 L) was packed in a glass column (φ44 mm × 100 cm) and washed with ultrapure water (2.0 L) followed by 1M HCl (4.2 L) and additional ultrapure water (2.5 L). Aqueous Me_4_NOH solution (2.5 wt%, 4.2 L) and ultrapure water (4.0 L) were successively passed through the column to adjust the resin to a Me_4_N type. Aqueous NaOCl·5H_2_O solution (8.0 wt% as available chlorine) was then passed through the column to afford the aqueous TMAOCl solution.

### 3.5. Characterization Data

*Chloro-1-phenylhexan-1-one* (**2a**) [26]: Silica-gel column chromatography (hexane/AcOEt = 97:3) gave 89 mg of **2a** (0.42 mmol, 83%) as a colorless oil. ^1^H NMR (400 MHz, CDCl_3_): *δ* 7.97−7.95 (m, 2H), 7.58−7.55 (m, 1H), 7.48−7.45 (m, 2H), 3.56 (t, *J* = 6.6 Hz, 2H), 3.00 (t, *J* = 7.4 Hz, 2H), 1.86−1.76 (m, 4H), 1.56−1.52 (m, 2H); ^13^C{^1^H} NMR (100 MHz, CDCl_3_): *δ* 200.0, 136.9, 133.0, 128.6, 128.0, 44.9, 38.3, 32.4, 26.6, 23.4; LRMS (ESI) *m*/*z*: 211 [M + H]^+^.
*6-Chloro-1-(4-chlorophenyl)hexan-1-one* (**2b**) [22]: Silica-gel column chromatography (hexane/AcOEt = 97:3) gave 103 mg of **2b** (0.42 mmol, 84%) as a colorless oil. ^1^H NMR (400 MHz, CDCl_3_): *δ* 7.91–7.88 (m, 2H), 7.46–7.42 (m, 2H), 3.56 (t, *J* = 6.6 Hz, 2H), 2.97 (t, *J* = 6.8 Hz, 2H), 1.85–1.75 (m, 4H), 1.56–1.53 (m, 2H); ^13^C{^1^H} NMR (100 MHz, CDCl_3_): *δ* 198.7, 139.4, 135.2, 129.4, 128.9, 44.8, 38.2, 32.4, 26.5, 23.3; LRMS (ESI) *m*/*z*: 245 [M + H]^+^.
*6-Chloro-1-(3-chlorophenyl)hexan-1-one* (**2c**): Silica-gel column chromatography (hexane/AcOEt = 97:3) gave 103 mg of **2c** (0.42 mmol, 84%) as a colorless oil. ^1^H NMR (400 MHz, CDCl_3_): *δ* 7.92 (s, 1H), 7.83 (d, *J* = 7.6 Hz, 1H), 7.55–7.51 (m, 1H), 7.43–7.39 (m, 1H), 3.56 (t, *J* = 6.6 Hz, 2H), 2.97 (t, *J* = 7.4 Hz, 2H), 1.86–1.75 (m, 4H), 1.56–1.52 (m, 2H); ^13^C{^1^H} NMR (100 MHz, CDCl_3_): *δ* 198.5, 139.8, 132.5, 128.4, 117.9, 116.3, 44.7, 38.6, 32.3, 26.4, 23.1; IR (ATR): 2939, 2865, 1686, 1570, 1420, 1206 cm^−1^; HRMS (ESI) *m*/*z*: [M + H]^+^ calculated for C_12_H_15_Cl_2_O 246.1529, found 246.1530.
*6-Chloro-1-(4-trifluoromethylphenyl)hexan-1-one* (**2d**): Silica-gel column chromatography (hexane/AcOEt = 97:3) gave 96 mg of **2d** (0.35 mmol, 69%) as a colorless oil. ^1^H NMR (400 MHz, CDCl_3_): *δ* 8.06 (d, *J* = 8.8 Hz, 2H), 7.73 (d, *J* = 8.0 Hz, 2H), 3.57 (t, *J* = 6.4 Hz, 2H), 3.03 (t, *J* = 7.2 Hz, 2H), 1.86–1.77 (m, 4H), 1.57–1.53 (m, 2H); ^13^C{^1^H} NMR (100 MHz, CDCl_3_): *δ* 198.9, 139.5, 134.3 (q, *J* = 32.4 Hz), 128.3, 125.7, 123.6 (q, *J* = 270.7 Hz), 44.8, 38.6, 32.4, 26.4, 23.1; ^19^F NMR (376 MHz, CDCl_3_): *δ* −66.3; IR (ATR): 2941, 2868, 1690, 1409, 1322, 1125, 1065 cm^−1^; HRMS (ESI) *m*/*z*: [M + H]^+^ calculated for C_13_H_15_ClF_3_O 279.7058, found 279.7057.
*4-(5-Chloropentanoyl)benzonitrile* (**2e**): Silica-gel column chromatography (hexane/AcOEt = 97:3) gave 84 mg of **2e** (0.36 mmol, 71%) as a colorless oil. ^1^H NMR (400 MHz, CDCl_3_): *δ* 8.04 (d, *J* = 8.8 Hz, 2H), 7.78 (d, *J* = 8.4 Hz, 2H), 3.56 (t, *J* = 6.4 Hz, 2H), 3.01 (t, *J* = 7.2 Hz, 2H), 1.86–1.77 (m, 4H), 1.57–1.55 (m, 2H); ^13^C{^1^H} NMR (100 MHz, CDCl_3_): *δ* 198.5, 139.8, 132.5, 128.4, 117.9, 116.3, 44.7, 38.6, 32.3, 26.4, 23.1; IR (ATR): 2947, 2866, 2229, 1695, 1402, 1272, 1191 cm^−1^; HRMS (ESI) *m*/*z*: [M + H]^+^ calculated for C_13_H_15_ClNO 236.7173, found 236.7172.
*6-Chloro-1-(4-methylphenyl)hexan-1-one* (**2f**) [23]: Silica-gel column chromatography (hexane/AcOEt = 97:3) gave 94 mg of **2f** (0.42 mmol, 84%) as a pale yellow solid. ^1^H NMR (400 MHz, CDCl_3_): *δ* 7.87–7.84 (m, 2H), 7.27–7.25 (m, 2H), 3.55 (t, *J* = 6.8 Hz, 2H), 2.97 (t, *J* = 7.4 Hz, 2H), 2.41 (s, 3H), 1.85–1.77 (m, 4H), 1.56–1.53 (m, 2H); ^13^C{^1^H} NMR (100 MHz, CDCl_3_): *δ* 199.7, 143.7, 134.5, 129.2, 128.1, 44.9, 38.2, 32.5, 26.6, 23.5, 21.6; LRMS (ESI) *m*/*z*: 225 [M + H]^+^.
*3-(2-Chloroethoxy)-1-phenyl-1-propanone* (**2g**): Silica-gel column chromatography (hexane/AcOEt = 97:3) gave 54 mg of **2g** (0.26 mmol, 51%) as a colorless oil. ^1^H NMR (400 MHz, CDCl_3_): *δ* 7.98–7.95 (m, 2H), 7.57–7.49 (m, 1H), 7.49–7.45 (m, 2H), 3.97–3.94 (m, 2H), 3.76–3.74 (m, 2H), 3.63–3.60 (m, 2H), 3.31–3.27 (m, 2H); ^13^C{^1^H} NMR (100 MHz, CDCl_3_): *δ* 198.0, 136.8, 133.2, 128.6, 128.0, 71.2, 66.3, 42.7, 38.6; IR (ATR): 2961, 2873, 1661, 1596, 1446, 1213, 1116 cm^−1^; HRMS (ESI) *m*/*z*: [M + H]^+^ calculated for C_11_H_14_ClO_2_ 213.6807, found 213.6807.
*6-Chloro-4-methyl-1-phenyl-1-hexanone* (**2i**) [23]: Silica-gel column chromatography (hexane/toluene = 4/6) gave 58 mg of **2i** (0.26 mmol, 52%) as a colorless oil. ^1^H NMR (400 MHz, CDCl_3_): *δ* 7.98–7.95 (m, 2H), 7.57–7.55 (m, 1H), 7.49–7.45 (m, 2H), 3.63–3.55 (m, 2H), 3.03–2.97 (m, 2H), 1.84–1.76 (m, 3H), 1.67–1.58 (m, 2H), 0.97 (d, *J* = 6.8 Hz, 3H); ^13^C{^1^H} NMR (100 MHz, CDCl_3_): *δ* 200.2, 136.9, 133.0, 128.6, 128.0, 43.0, 39.5, 36.0, 30.7, 30.1, 18.9; LRMS (ESI) *m*/*z*: 225 [M + H]^+^.
*5-Chloro-1-phenylpentan-1-one* (**2j**) [26]: Silica-gel column chromatography (hexane/AcOEt = 97:3) gave 53 mg of **2j** (0.27 mmol, 54%) as a colorless oil. ^1^H NMR (400 MHz, CDCl_3_): *δ* 7.97–7.95 (m, 2H), 7.57–7.56 (m, 1H), 7.49–7.45 (m, 2H), 3.59 (t, *J* = 6.4 Hz, 2H), 3.02 (t, *J* = 6.8 Hz, 2H), 1.91–1.89 (m, 4H); ^13^C{^1^H} NMR (100 MHz, CDCl_3_): *δ* 199.6, 136.8, 133.1, 128.6, 128.0, 44.7, 37.5, 32.0, 21.5; LRMS (ESI) *m*/*z:* 197 [M + H]^+^.
*7-Chloro-1-phenylheptan-1-one* (**2k**) [26]: Silica-gel column chromatography (hexane/AcOEt = 97:3) gave 81 mg of **2k** (0.36 mmol, 72%) as a colorless oil. ^1^H NMR (400 MHz, CDCl_3_): *δ* 7.97–7.95 (m, 2H), 7.58–7.54 (m, 1H), 7.48–7.44 (m, 2H), 3.54 (t, *J* = 6.8 Hz, 2H), 2.98 (t, *J* = 7.2 Hz, 2H), 1.82–1.75 (m, 4H), 1.50–1.42 (m, 4H); ^13^C{^1^H} NMR (100 MHz, CDCl_3_): *δ* 200.3, 137.0, 132.9, 128.6, 128.0, 45.0, 38.4, 32.4, 28.5, 26.7, 24.0; LRMS (ESI) *m*/*z*: 225 [M + H]^+^.
*8-Chloro-1-phenyloctan-1-one* (**2l**) [26]: Silica-gel column chromatography (hexane/AcOEt = 97:3) gave 50 mg of **2l** (0.36 mmol, 42%) as a colorless oil. ^1^H NMR (400 MHz, CDCl_3_): *δ* 7.98–7.95 (m, 2H), 7.59–7.52 (m, 1H), 7.49–7.44 (m, 2H), 3.53 (t, *J* = 6.8 Hz, 2H), 2.97 (t, *J* = 7.2 Hz, 2H), 1.80–1.73 (m, 8H), 0.93–0.82 (m, 3H); ^13^C{^1^H} NMR (100 MHz, CDCl_3_): *δ* 200.4, 137.0, 132.9, 128.5, 128.0, 45.1, 38.5, 32.5, 29.1, 28.7, 26.7, 24.2; LRMS (ESI) *m*/*z*: 239 [M + H]^+^.

## 4. Conclusions

In conclusion, we developed the C–C bond cleavage of *tert*-cycloalkanols for the direct approach for ω-chloroalkyl aryl ketones using TMAOCl as an oxidant. TMAOCl demonstrated a higher reactivity rather than the combination of NaOCl/Me_4_NCl, which might indicate the usefulness of TMAOCl as not only a hypochlorite but also as a phase-transfer reagent. In this reaction, we successfully obtained a variety of ω-chloroalkyl aryl ketones in good to high yields, even on a gram scale.

## Figures and Tables

**Table 1:** Optimization of reaction conditions.

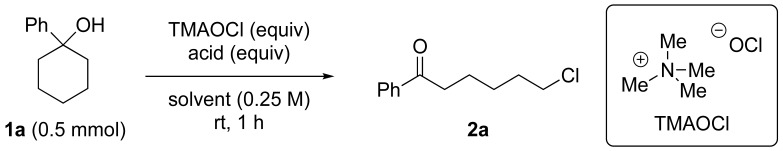				
Entry	TMAOCl (Equiv)	Acid (Equiv)	Solvent	Yield (%) *^a^*
1	1.5	35% HCl (1.5)	CH_2_Cl_2_	47
2	1.5	TFA (1.5)	CH_2_Cl_2_	77
3	1.5	AcOH (1.5)	CH_2_Cl_2_	79
4	1.5	H_3_PO_4_ (0.75)	CH_2_Cl_2_	76
5	1.5	NaH_2_PO_4_ (1.5)	CH_2_Cl_2_	75
6	1.5	AcOH (2.0)	CH_2_Cl_2_	85
7	1.5	AcOH (2.25)	CH_2_Cl_2_	83
8	2.0	AcOH (2.0)	CH_2_Cl_2_	70
9 *^b^*	1.5	AcOH (2.0)	CH_2_Cl_2_	85
10 *^c^*	1.5	AcOH (2.0)	CH_2_Cl_2_	85 (83)
11 *^c,d^*	1.5	AcOH (2.0)	CH_2_Cl_2_	84
12 *^c^*	1.5	AcOH (2.0)	ClPh	70
13 *^c^*	1.5	AcOH (2.0)	AcOEt	67
14 *^c^*	1.5	AcOH (2.0)	MeCN	62
15 *^c,e^*	-	AcOH (2.0)	CH_2_Cl_2_	56
16 *^c,f^*	-	AcOH (2.0)	CH_2_Cl_2_	74
17 *^c,g^*	-	AcOH (2.0)	CH_2_Cl_2_	79
18	1.5	-	CH_2_Cl_2_	trace
19 *^h^*	1.5	AcOH (2.0)	CH_2_Cl_2_	77

*^a^* Determined by ^1^H NMR analysis using maleic acid as an internal standard. Isolated yield is given in parentheses. *^b^* 0.5 M. *^c^* 1.0 M. *^d^* 4 h. *^e^* NaOCl·5H_2_O (1.5 equiv). *^f^* NaOCl·5H_2_O (1.5 equiv), Me_4_NCl (1.5 equiv). *^g^* NaOCl·5H_2_O (2.0 equiv), Me_4_NCl (2.0 equiv). *^h^* Under N_2_ atmosphere.

## Data Availability

The data presented in this study are available in Supplementary Materials.

## Supplementary Materials

The following supporting information can be downloaded at: https://www.mdpi.com/article/10.3390/molecules29081874/s1, Copies of ^1^H, ^13^C{^1^H}, and ^19^F NMR spectra of compounds **2a**–**2g** and **2i**–**2l**.
