# Supplementary material for: Synthesis of ω-Chloroalkyl Aryl Ketones via C–C Bond Cleavage of tert-Cycloalkanols with Tetramethylammonium Hypochlorite
Source: Molecules. 2024 Apr 19;29(8):1874. doi: 10.3390/molecules29081874 (PMC11055113; doi:10.3390/molecules29081874)
Supplement: Supplementary file 1 [file molecules-29-01874-s001.zip › molecules-2942314-supplementary.pdf]

Synthesis of ω-Chloroalkyl Aryl Ketones via C–C Bond Cleavage of *tert*-Cycloalkanols with Tetramethylammonium Hypochlorite

Natsumi Hanazawa, Masami Kuriyama,
Kosuke Yamamoto and Osamu Onomura*

Graduate School of Biomedical Sciences, Nagasaki University,
1-14 Bunkyo-Machi, Nagasaki 852-8521, Japan

* onomura@nagasaki-u.ac.jp

Supporting Information

Table of Contents

1. ^{1}H, $^{13}C\{^{1}H\}$, and ^{19}F NMR spectra S2-S13

1. ^{1}H, ^{13}C{^{1}H}, and ^{19}F NMR spectra

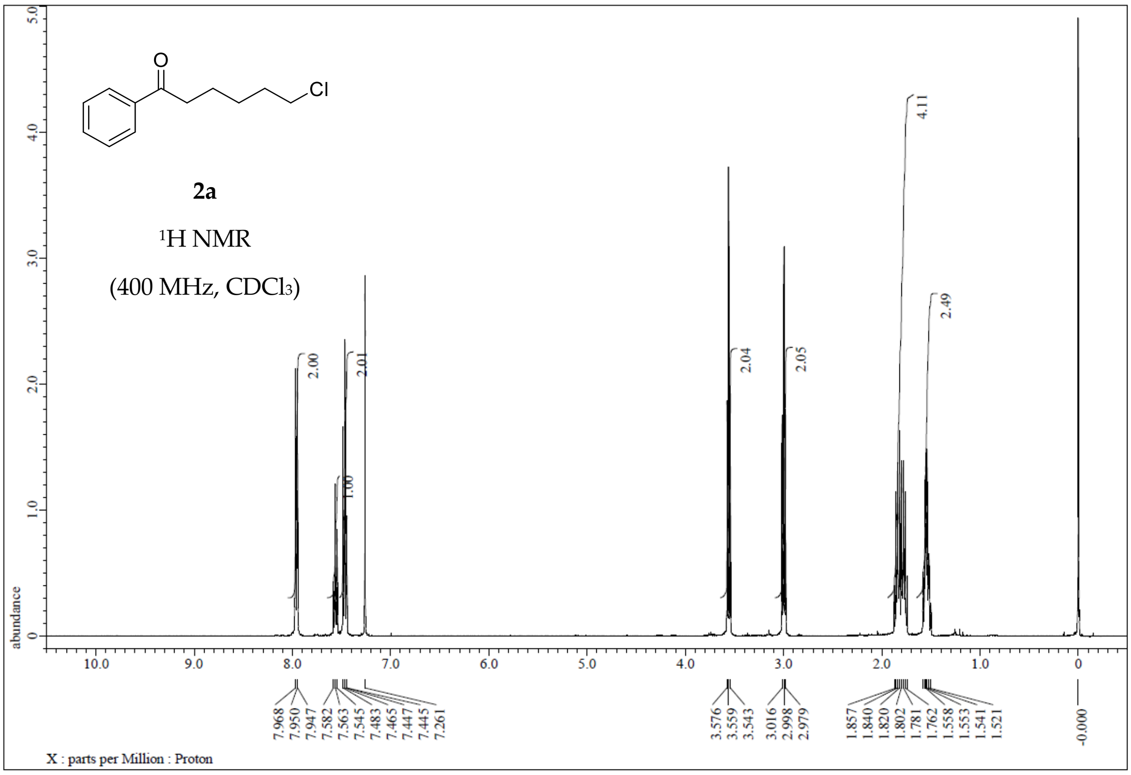

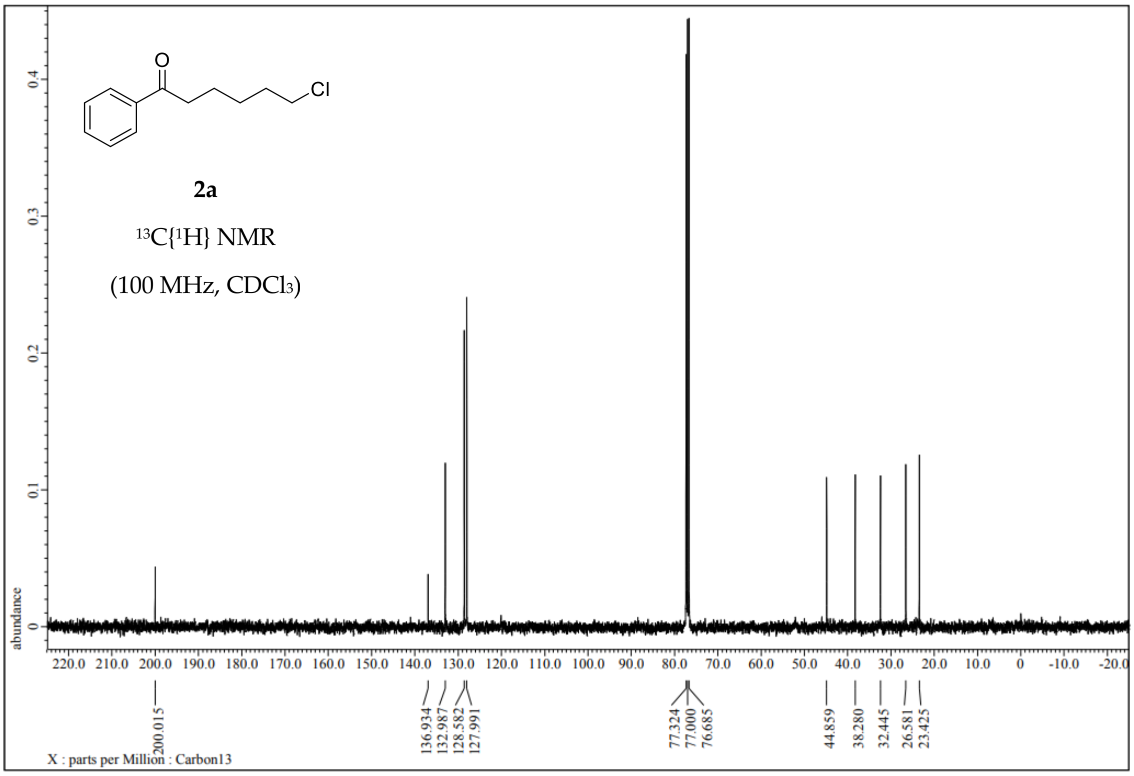

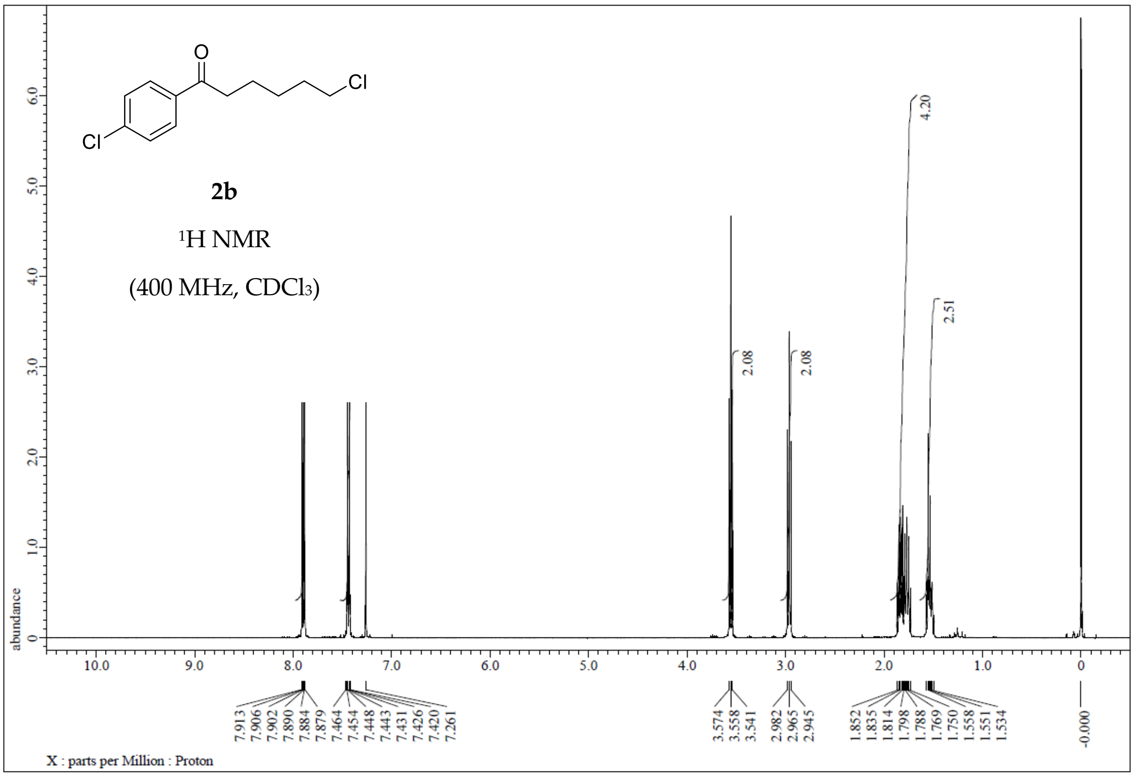
2b
^{1}H NMR
(400 MHz, CDCl3)
X : parts per Million : Proton
abundance

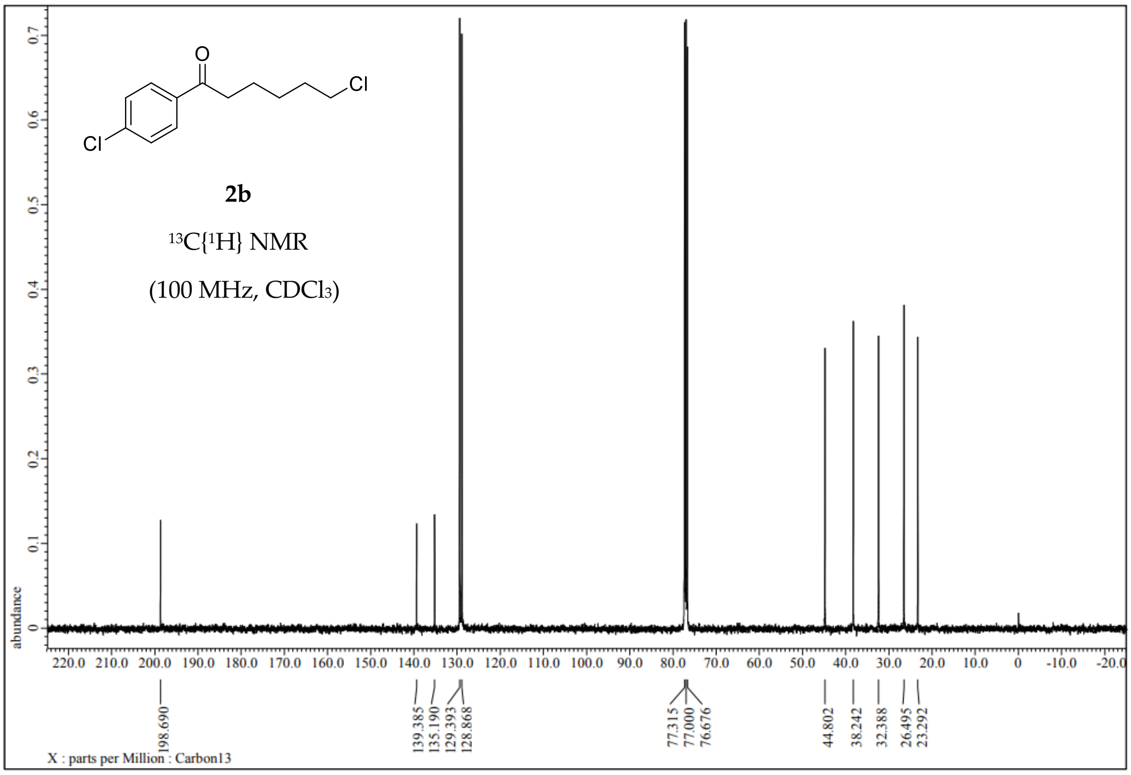
2b
^{13}C{^{1}H} NMR
(100 MHz, CDCl3)
X : parts per Million : Carbon13
abundance

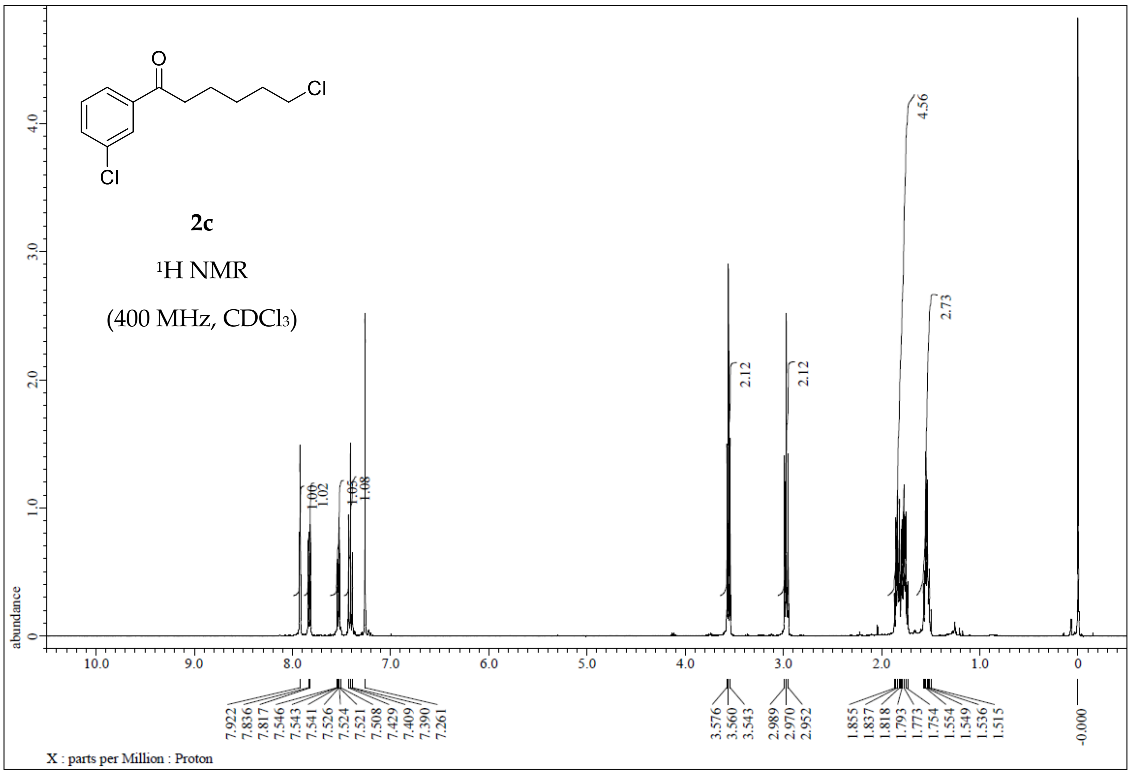
2c
¹H NMR
(400 MHz, CDCl3)
abundance
X : parts per Million : Proton
7.922 7.836 7.817 7.546 7.543 7.541 7.526 7.524 7.521 7.508 7.429 7.409 7.390 7.261
3.576 3.560 3.543
2.989 2.970 2.952
1.855 1.837 1.818 1.793 1.773 1.754 1.554 1.549 1.536 1.515
-0.000
1.00 1.02 1.05 1.08 2.12 2.12 4.56 2.73

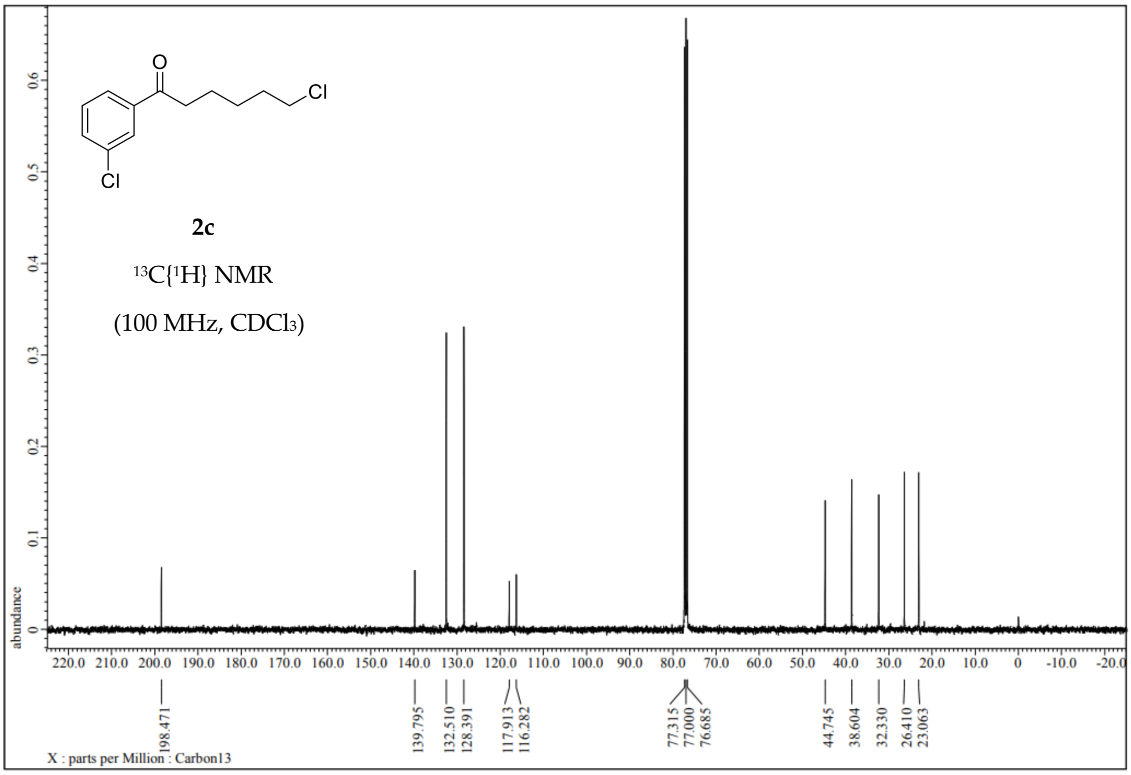
2c
¹³C{¹H} NMR
(100 MHz, CDCl3)
abundance
X : parts per Million : Carbon13
198.471
139.795
132.510
128.391
117.913
116.282
77.315
77.000
76.685
44.745
38.604
32.330
26.410
23.063

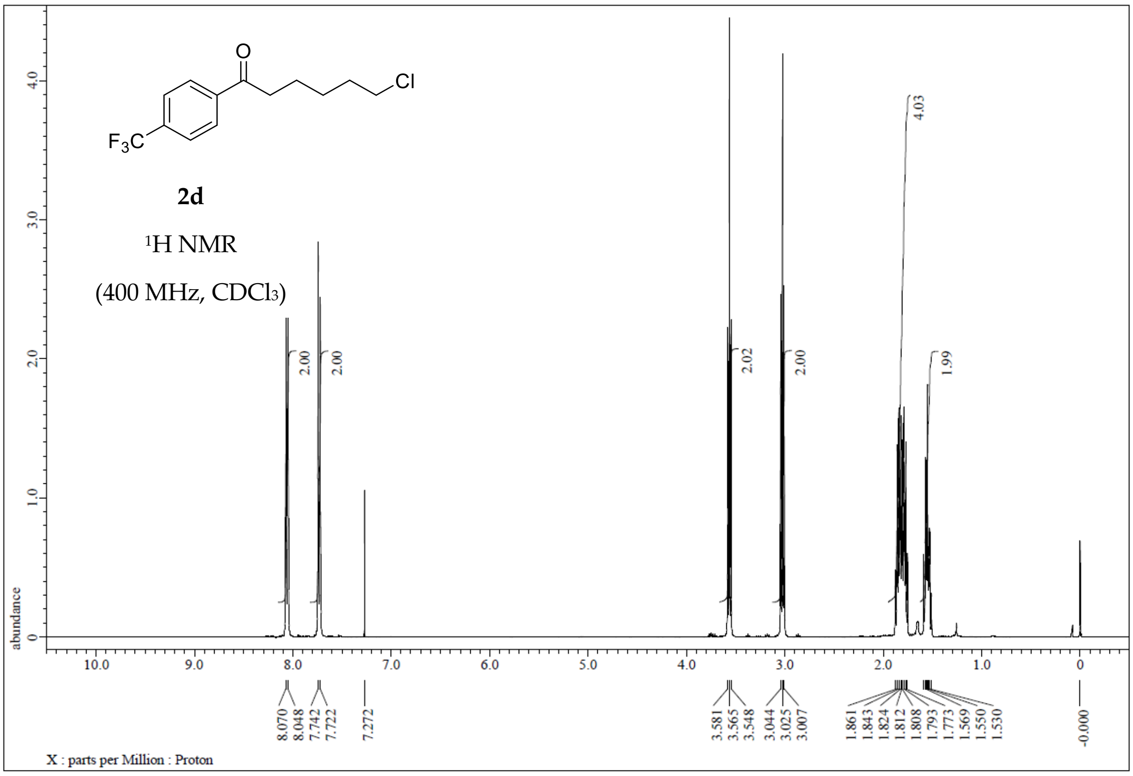
O
Cl
F_3C
2d
^{1}H NMR
(400 MHz, $CDCl_3$)
2.00
2.00
2.02
2.00
4.03
1.99
abundance
X : parts per Million : Proton
8.070
8.048
7.742
7.722
7.272
3.581
3.565
3.548
3.044
3.025
3.007
1.861
1.843
1.824
1.812
1.808
1.793
1.773
1.569
1.550
1.530
-0.000

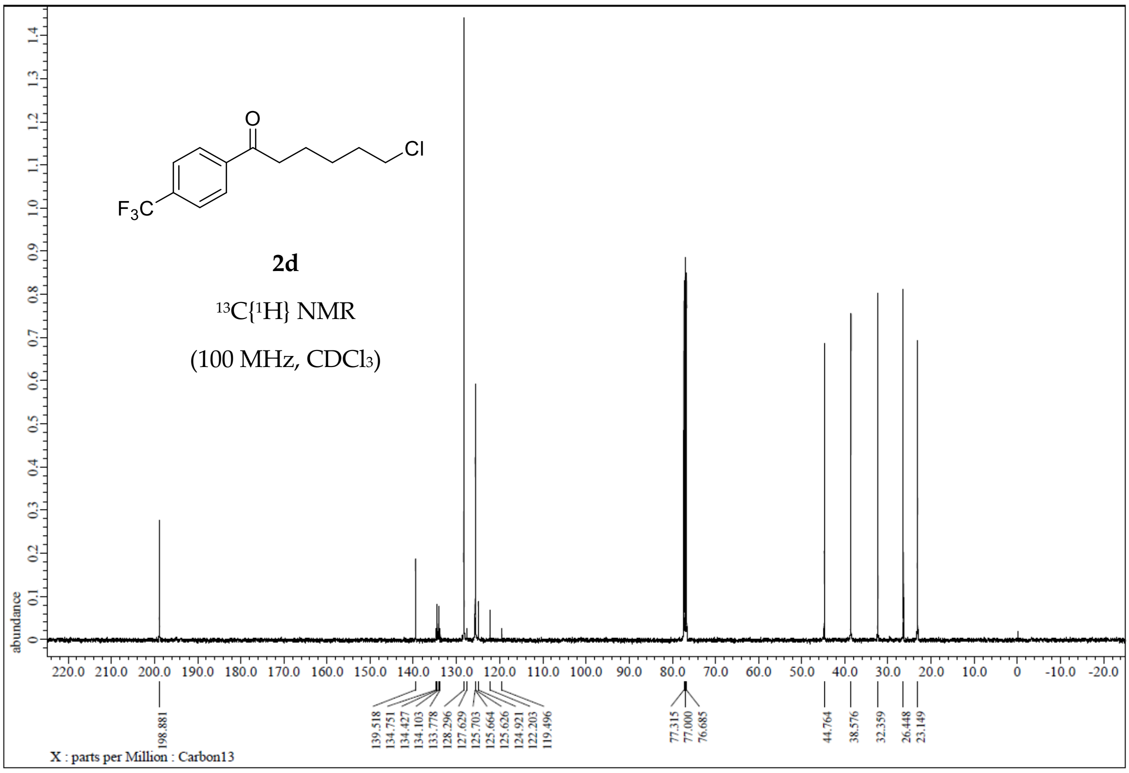
O
Cl
F_3C
2d
^{13}C{^{1}H} NMR
(100 MHz, $CDCl_3$)
abundance
X : parts per Million : Carbon13
198.881
139.518
134.751
134.427
134.103
133.778
128.296
127.629
125.703
125.664
125.626
124.921
122.203
119.496
77.315
77.000
76.685
44.764
38.576
32.359
26.448
23.149

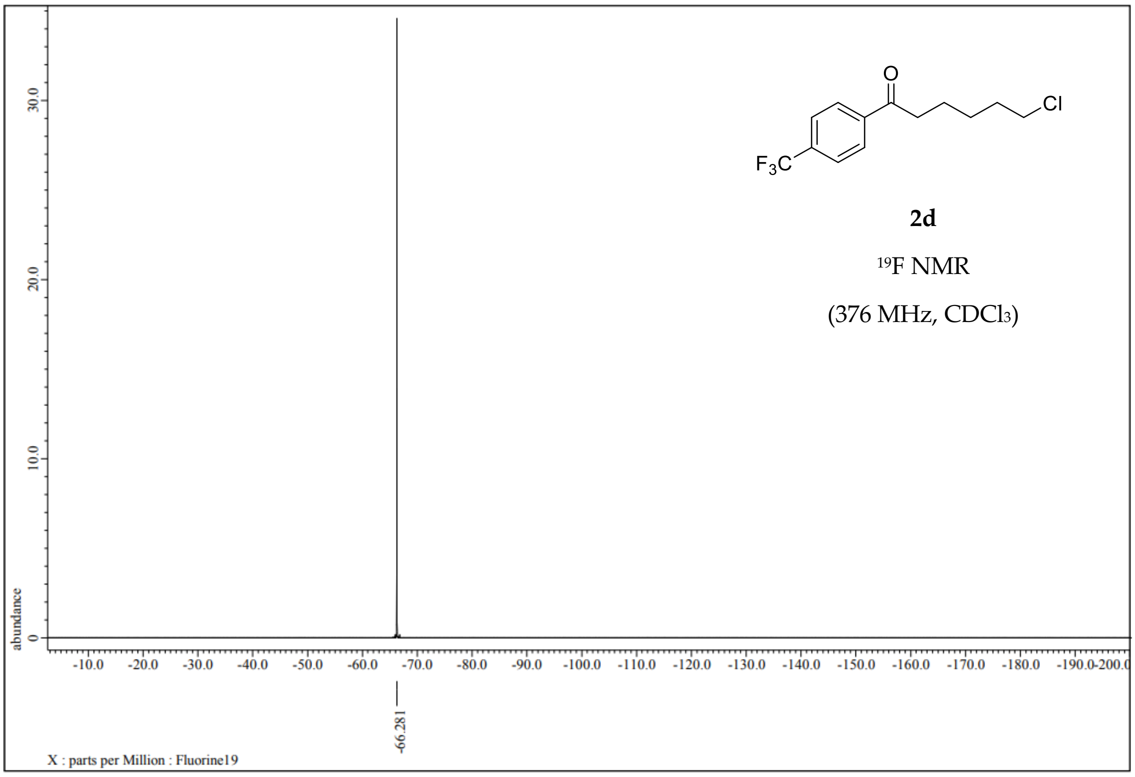

O
Cl
F_3C
2d
^{19}F NMR
(376 MHz, $CDCl_3$)
abundance
0
10.0
20.0
30.0
-10.0
-20.0
-30.0
-40.0
-50.0
-60.0
-70.0
-80.0
-90.0
-100.0
-110.0
-120.0
-130.0
-140.0
-150.0
-160.0
-170.0
-180.0
-190.0
-200.0
-66.281
X : parts per Million : Fluorine19

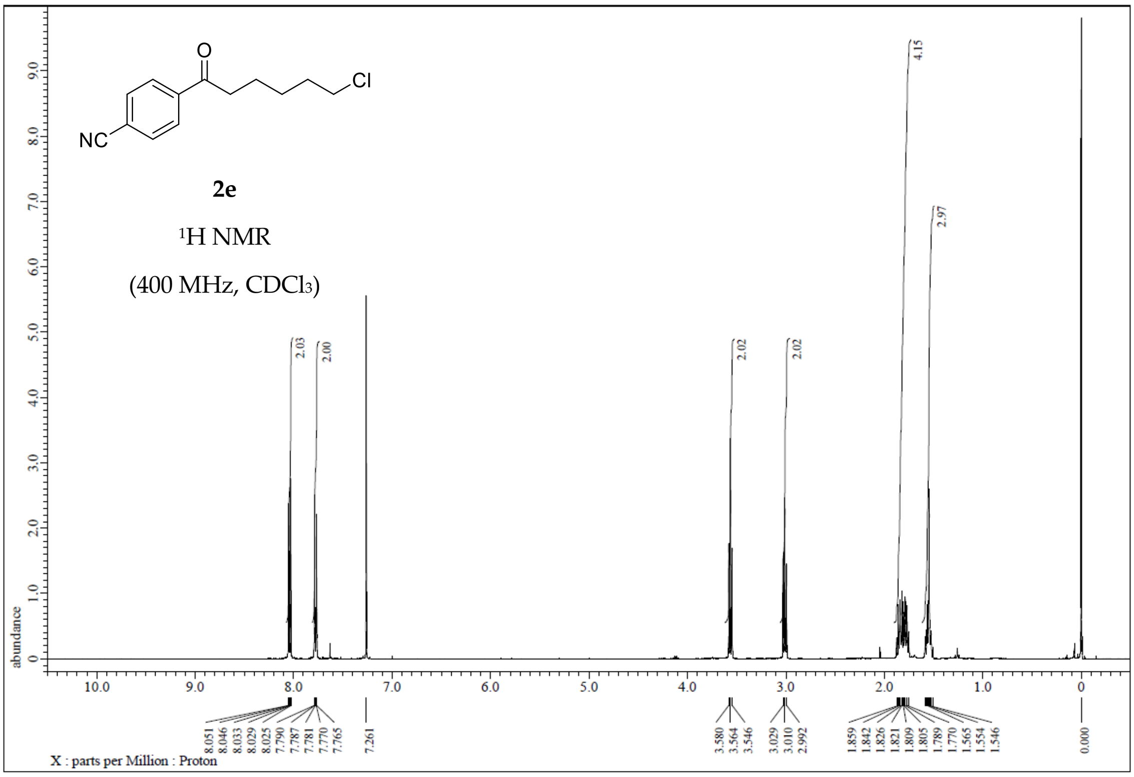
O
Cl
NC
2e
1H NMR
(400 MHz, CDCl3)
X : parts per Million : Proton
abundance

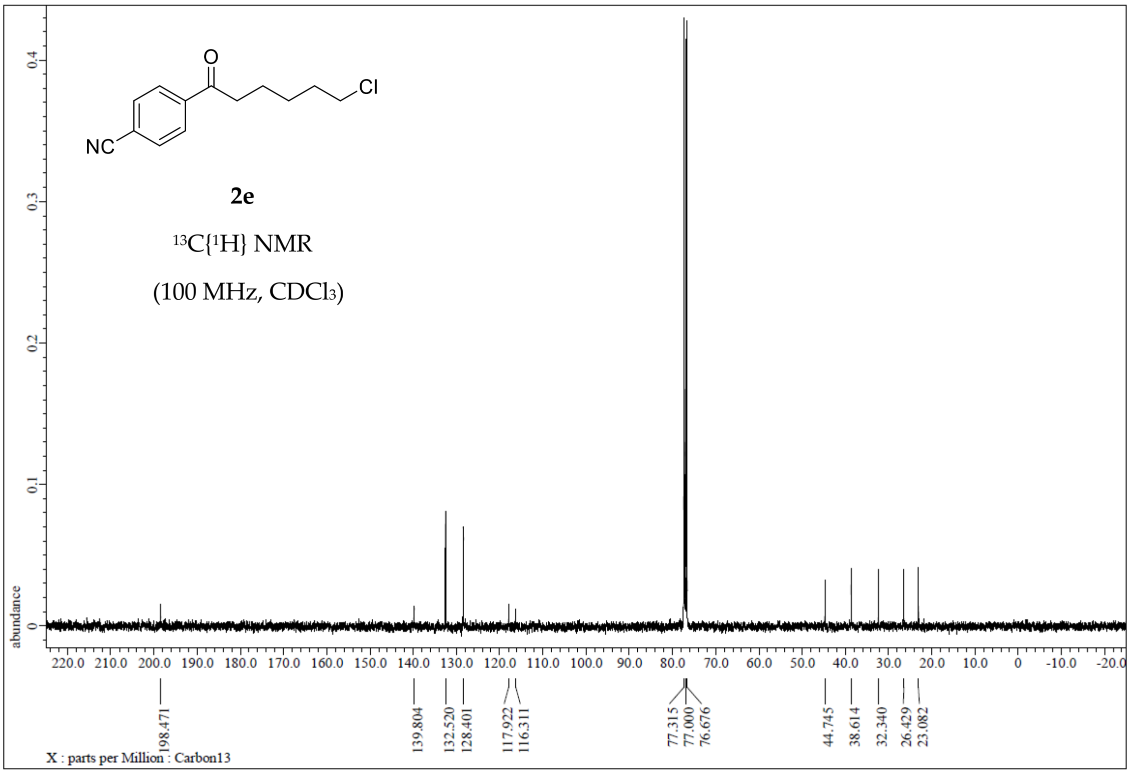
O
Cl
NC
2e
13C{1H} NMR
(100 MHz, CDCl3)
198.471
139.804
132.520
128.401
117.922
116.311
77.315
77.000
76.676
44.745
38.614
32.340
26.429
23.082
X : parts per Million : Carbon13
abundance

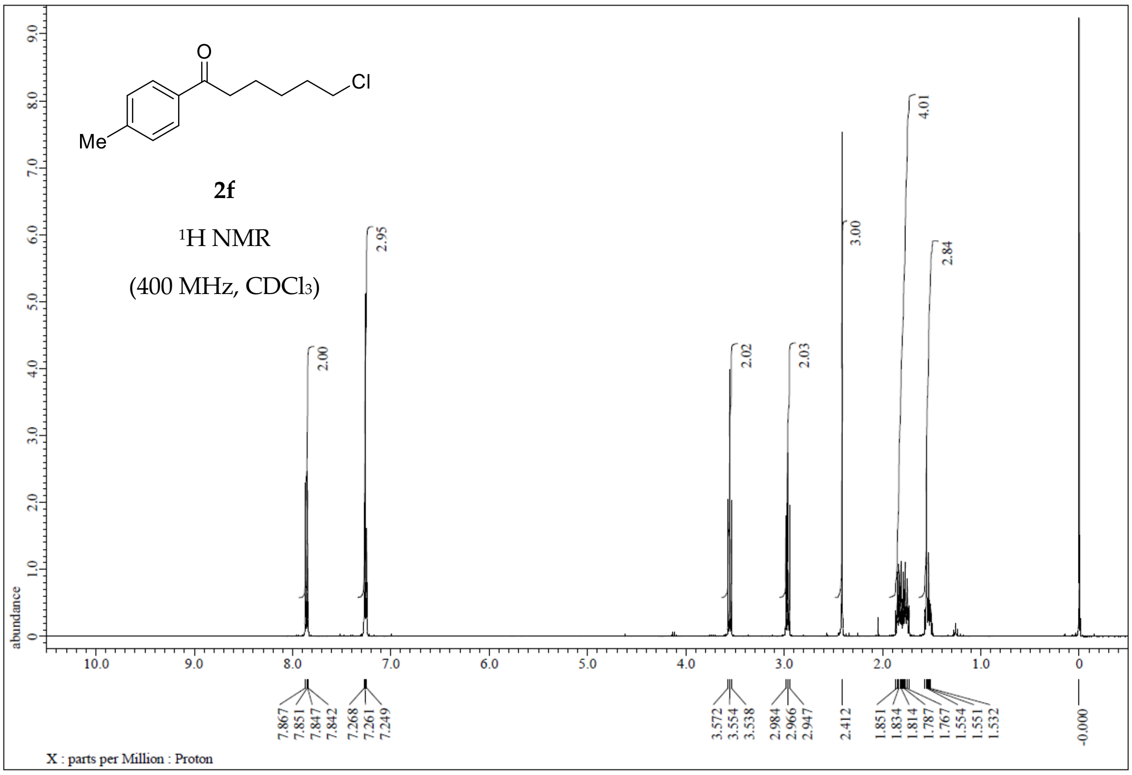
2f
1H NMR
(400 MHz, CDCl3)
X : parts per Million : Proton
abundance
7.867
7.851
7.847
7.842
7.268
7.261
7.249
3.572
3.554
3.538
2.984
2.966
2.947
2.412
1.851
1.834
1.814
1.787
1.767
1.554
1.551
1.532
-0.000
2.00
2.95
2.02
2.03
3.00
4.01
2.84

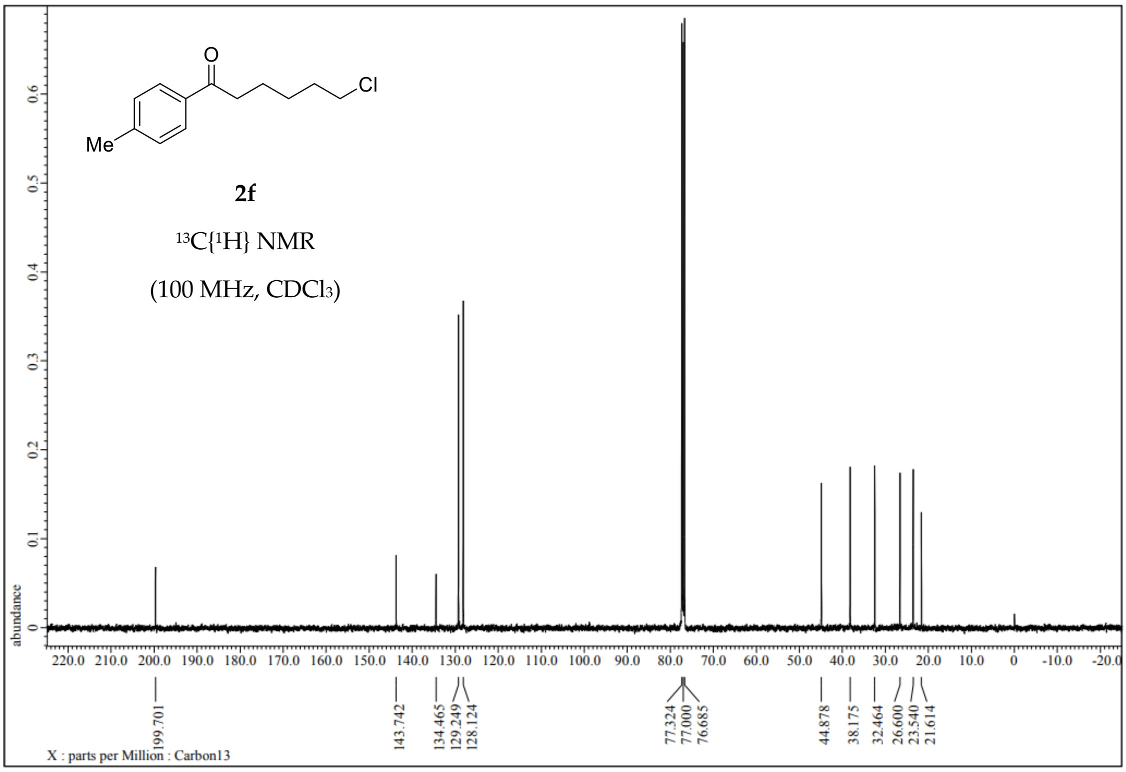
2f
13C{1H} NMR
(100 MHz, CDCl3)
X : parts per Million : Carbon13
abundance
199.701
143.742
134.465
129.249
128.124
77.324
77.000
76.685
44.878
38.175
32.464
26.600
23.540
21.614

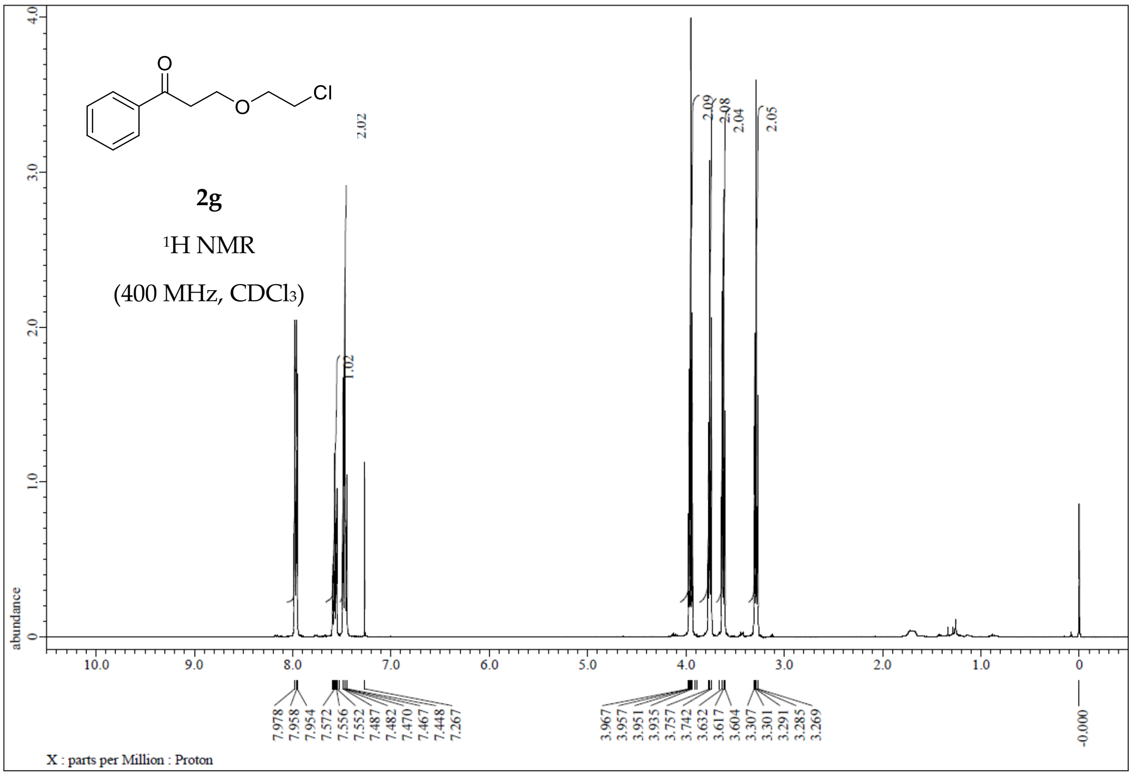
2g
^{1}H NMR
(400 MHz, CDCl$_3$)
X : parts per Million : Proton
abundance

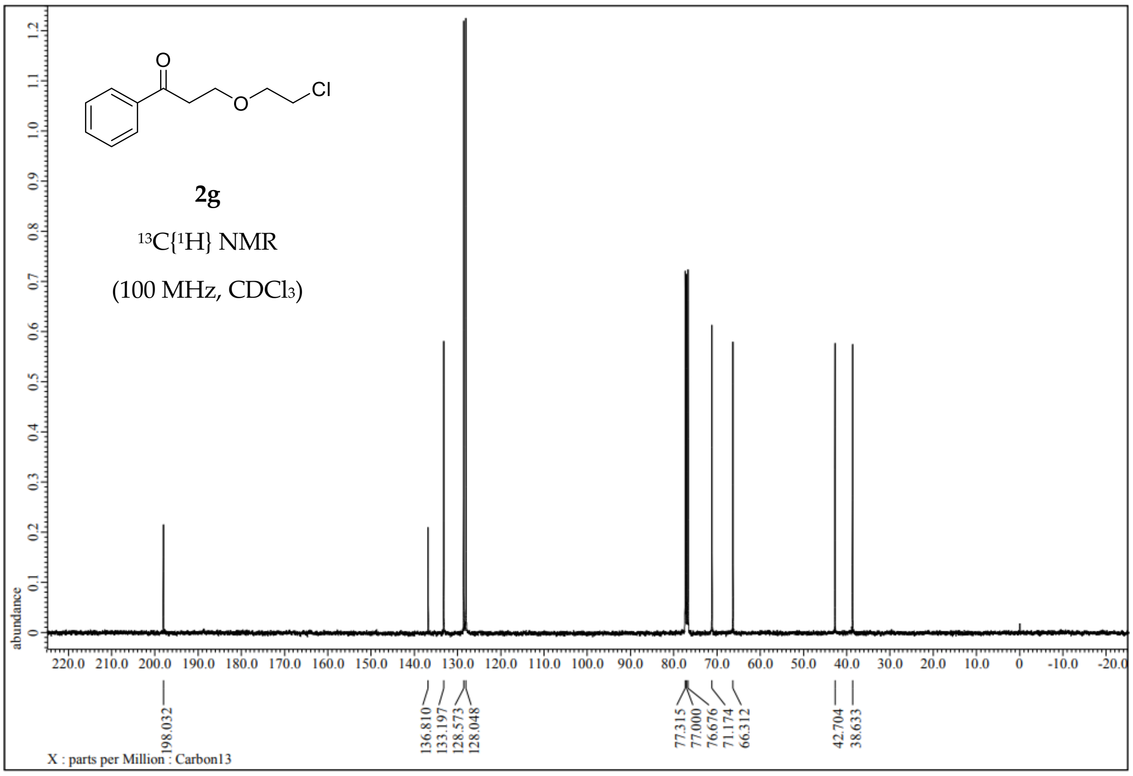
2g
^{13}C{^{1}H} NMR
(100 MHz, CDCl$_3$)
X : parts per Million : Carbon13
abundance

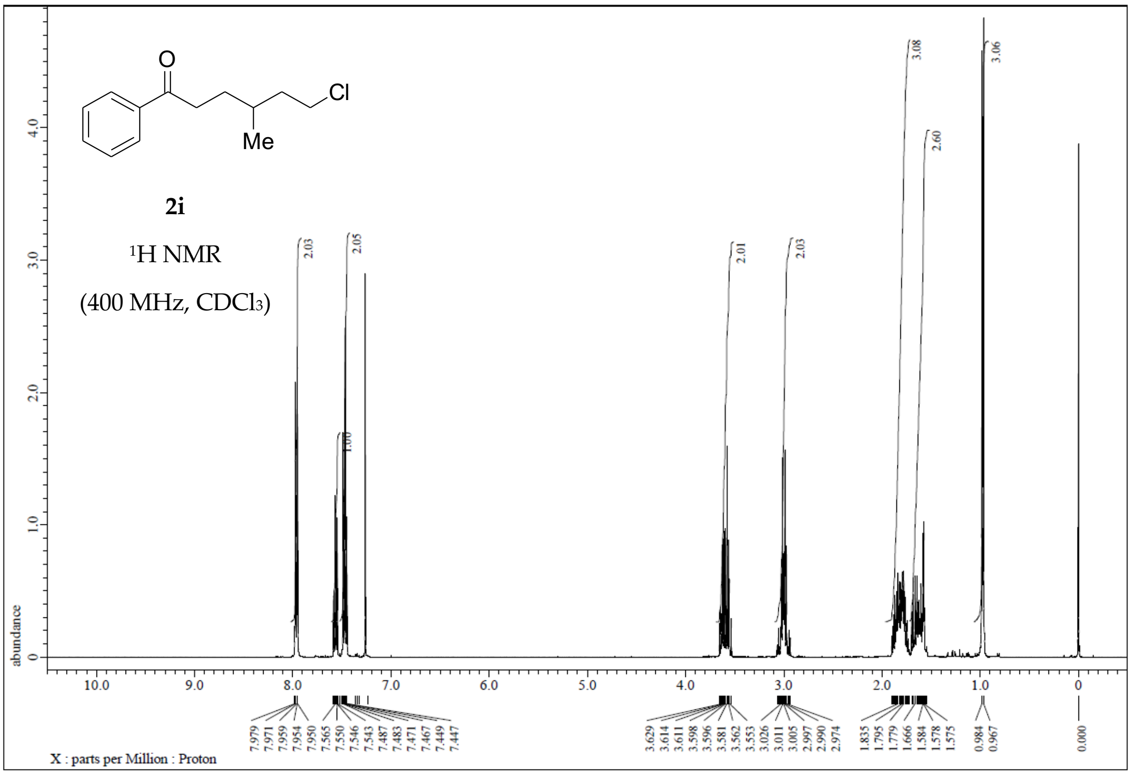
2i
^{1}H NMR
(400 MHz, CDCl$_3$)
X : parts per Million : Proton

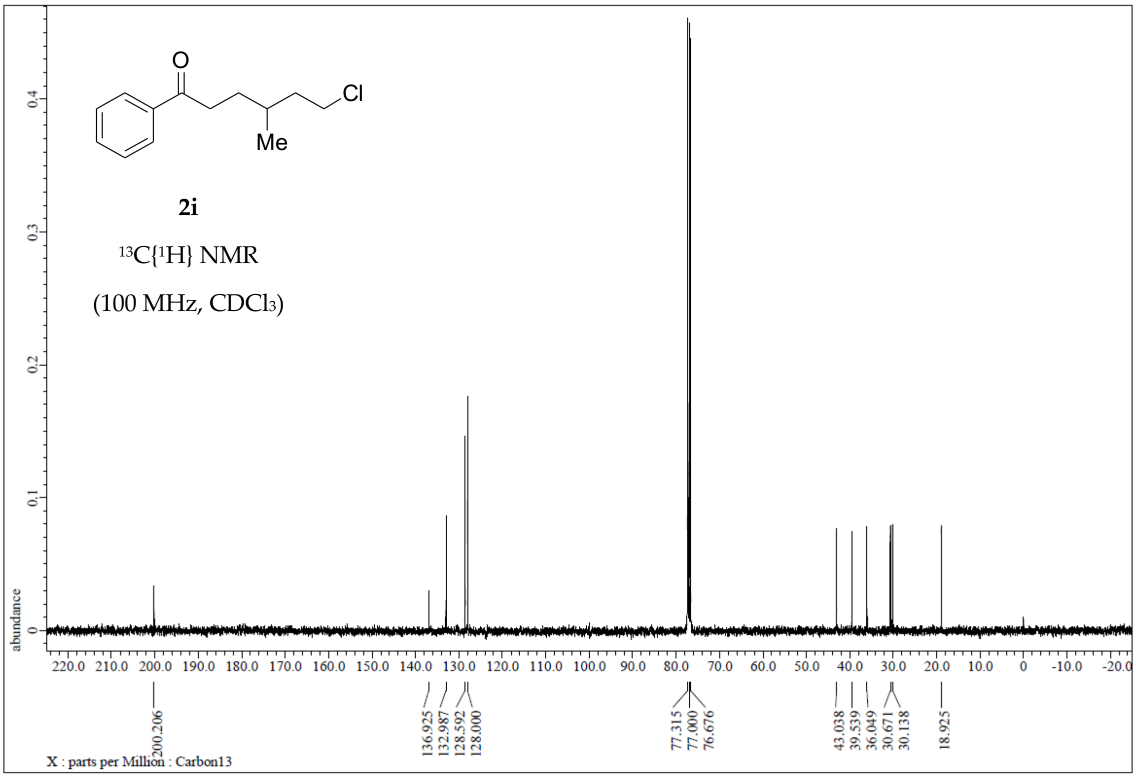
2i
^{13}C{^{1}H} NMR
(100 MHz, CDCl$_3$)
X : parts per Million : Carbon13

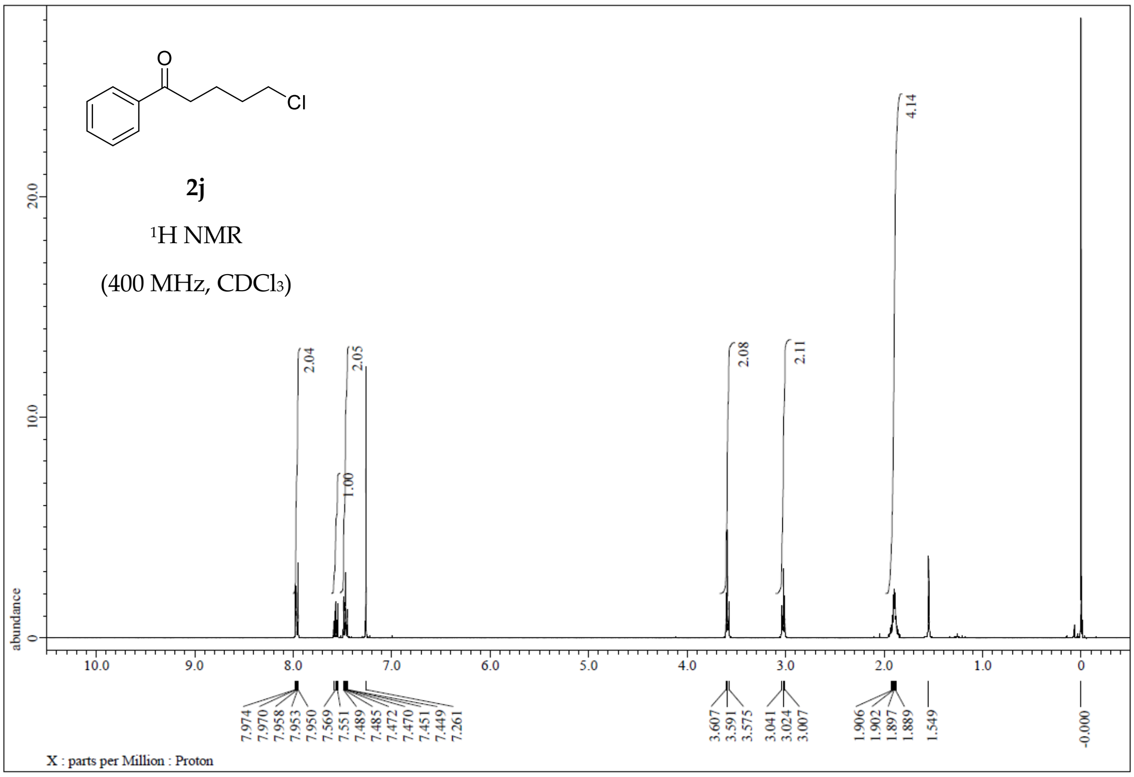
2j
^{1}H NMR
(400 MHz, CDCl$_3$)
X : parts per Million : Proton
abundance

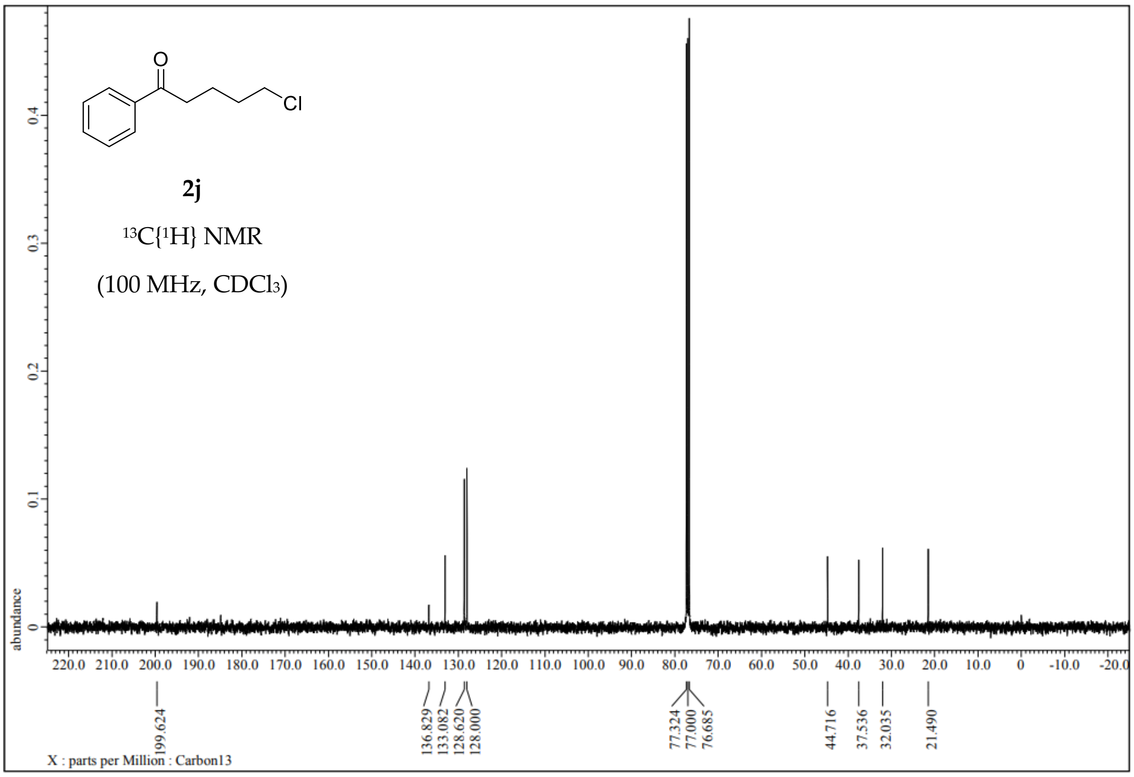
2j
^{13}C{^{1}H} NMR
(100 MHz, CDCl$_3$)
X : parts per Million : Carbon13
abundance

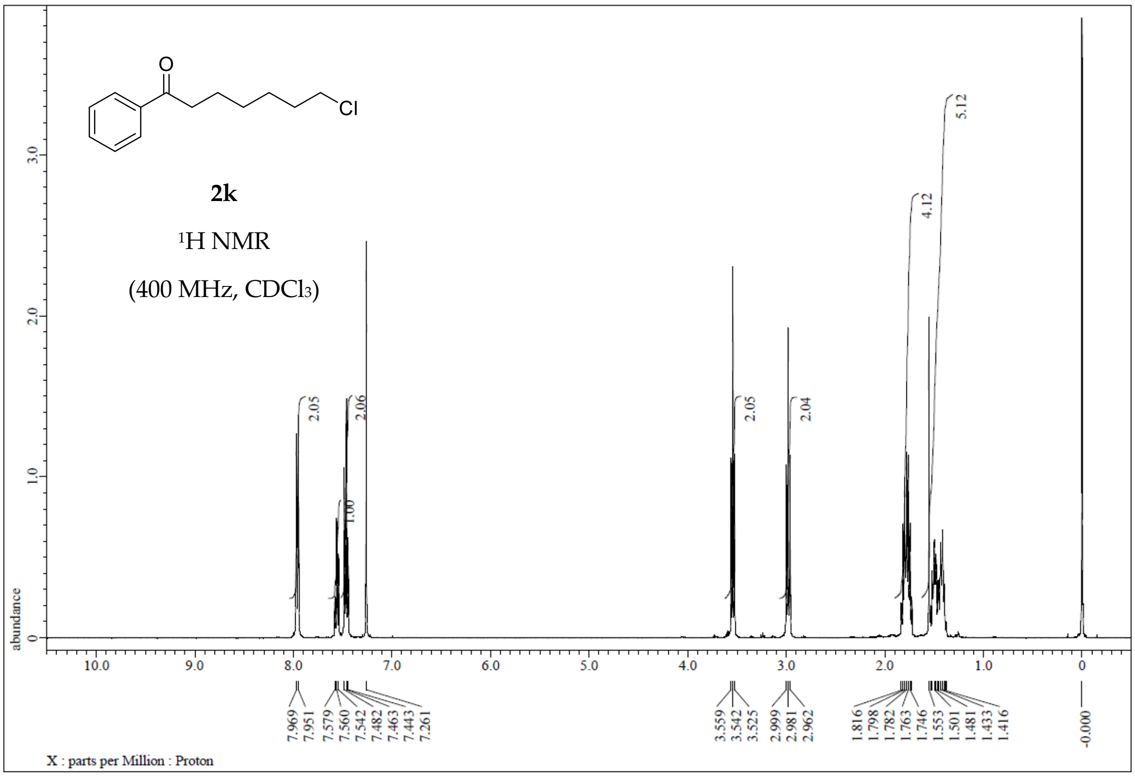
O
Cl
2k
1H NMR
(400 MHz, CDCl3)
2.05
2.06
1.00
2.05
2.04
4.12
5.12
abundance
10.0 9.0 8.0 7.0 6.0 5.0 4.0 3.0 2.0 1.0 0
7.969 7.951 7.579 7.560 7.542 7.482 7.463 7.443 7.261
3.559 3.542 3.525 2.999 2.981 2.962
1.816 1.798 1.782 1.763 1.746 1.553 1.501 1.481 1.433 1.416
-0.000
X : parts per Million : Proton

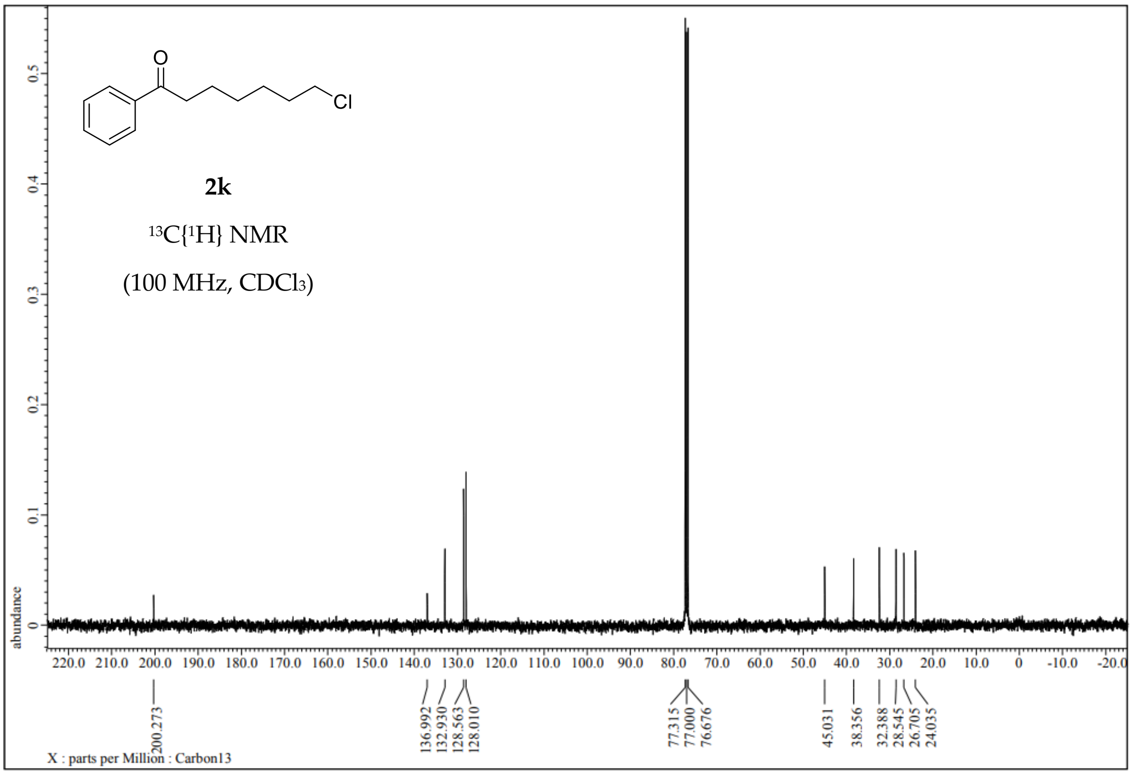
O
Cl
2k
13C{1H} NMR
(100 MHz, CDCl3)
abundance
220.0 210.0 200.0 190.0 180.0 170.0 160.0 150.0 140.0 130.0 120.0 110.0 100.0 90.0 80.0 70.0 60.0 50.0 40.0 30.0 20.0 10.0 0 -10.0 -20.0
200.273
136.992 132.930 128.563 128.010
77.315 77.000 76.676
45.031 38.356 32.388 28.545 26.705 24.035
X : parts per Million : Carbon13

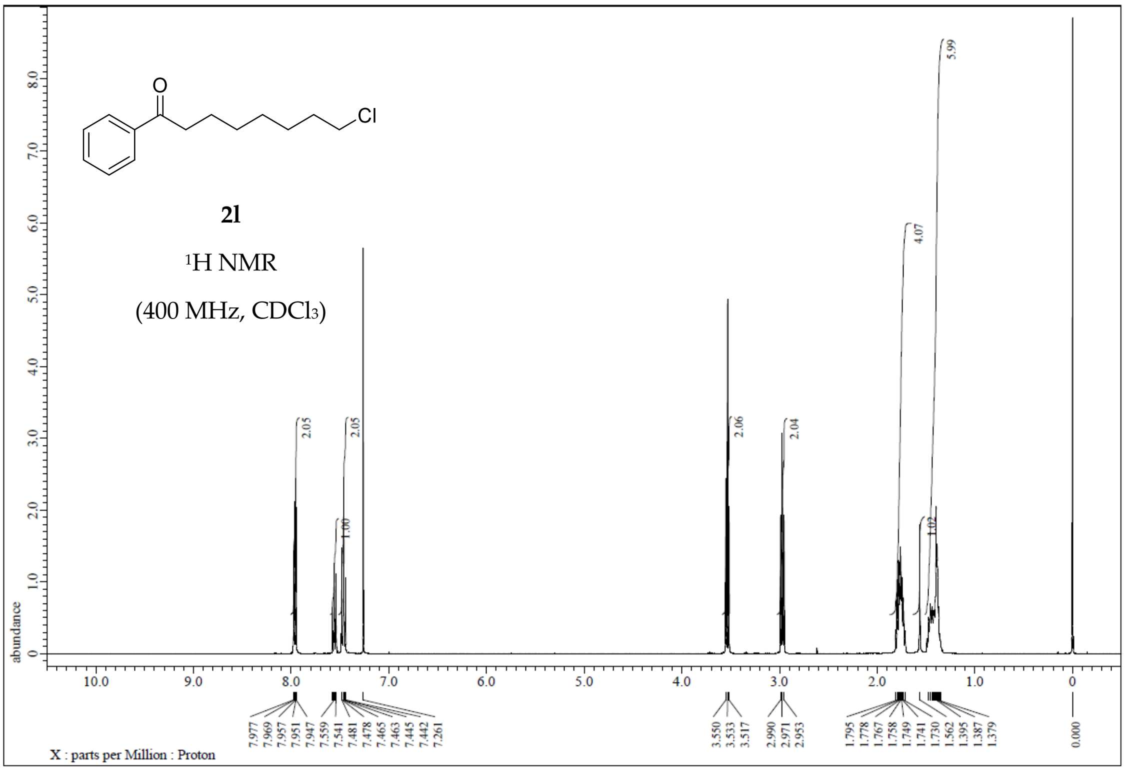
O
Cl
2l
1H NMR
(400 MHz, CDCl3)
abundance
X : parts per Million : Proton

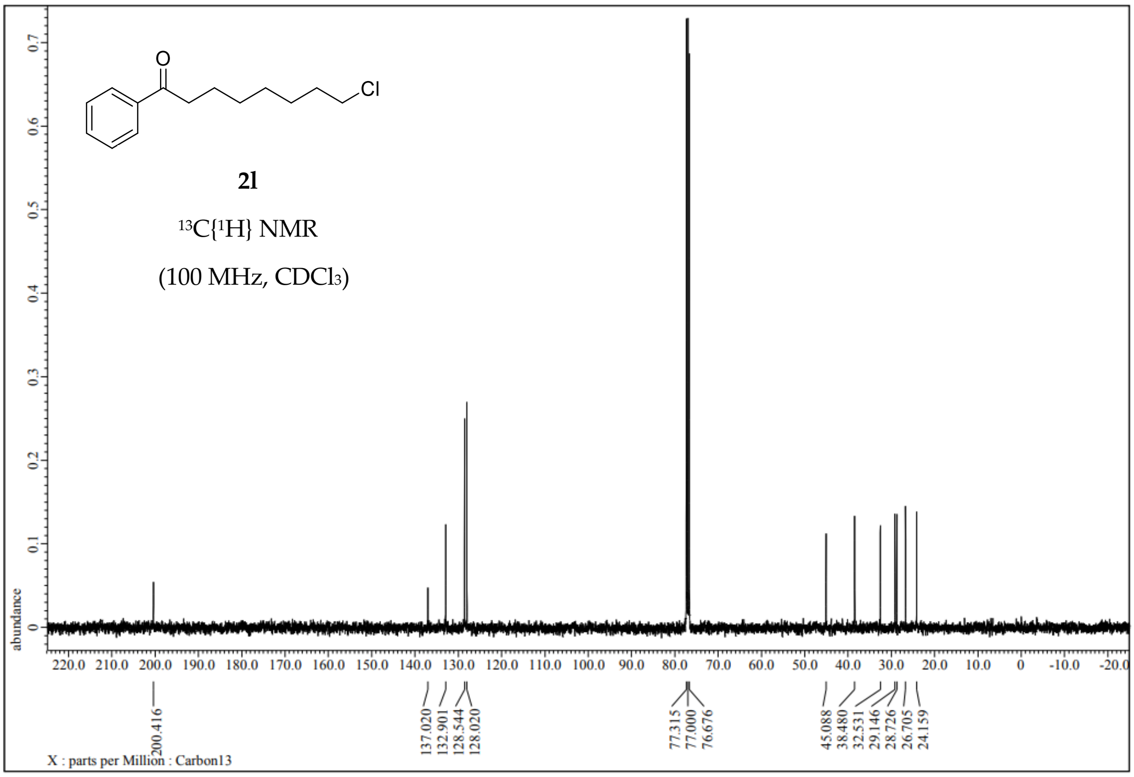
O
Cl
2l
13C{1H} NMR
(100 MHz, CDCl3)
abundance
X : parts per Million : Carbon13
